# Prevalence and risk factor analysis for the nonalcoholic fatty liver disease in patients with type 2 diabetes mellitus

**DOI:** 10.1097/MD.0000000000024940

**Published:** 2021-03-12

**Authors:** Qiumei Zhou, Yulong Wang, Jiajia Wang, Yating Liu, Dehui Qi, Wei Yao, Hui Jiang, Tingting Li, Kaiquan Huang, Wancun Zhang, Xingxing Huo

**Affiliations:** a Experimental Center of Clinical Research, The First Affiliated Hospital of Anhui University of Chinese Medicine; b Anhui Provincial Key Laboratory of Microbial Pest Control, Anhui Agricultural University; c Department of Ultrasonic Medicine; d Department of Laboratory Medicine, The First Affiliated Hospital of Anhui University of Chinese Medicine, Hefei; e Zhengzhou Antu Biological Engineering Co. LTD; f Department of Pediatric Oncology Surgery, Henan Provincial Key Laboratory of Children's Genetics and Metabolic Diseases, Children's Hospital Affiliated to Zhengzhou University, Zhengzhou, China.

**Keywords:** metabolism, non-alcoholic fatty liver disease, obesity, routine examination, screening, type-2 diabetes mellitus

## Abstract

Although non-alcoholic fatty liver disease (NAFLD) is strongly associated with type 2 diabetes mellitus (T2DM), the diagnosis of NAFLD for T2DM patients remains a challenge.

This study aimed to investigate the prevalence and risk factors for the NAFLD in T2DM outpatients.

This is a retrospective, cross-sectional study that included 2405 T2DM patients treated and admitted for glucose control into the Endocrinology Department of our hospital from April 2017 to March 2019. Using strict exclusion criteria, the target patients were screened and divided into two groups: NAFLD patients (study group) and non-NAFLD patients (control group). Subsequently, 34 factors were compared between the two groups. Furthermore, multivariate analysis of the NAFLD risk factors was performed using logistic regression. Finally, the diagnostic significance of individual biochemical predictors, as well as the combined predictive indicator (CPI), for NAFLD was estimated using receiver operating characteristic (ROC) curve analysis.

In this study, the overall prevalence of NAFLD in T2DM patients was 58.67%. Of the target patients, 17 factors were identified by univariate analysis to be associated with NAFLD, and 8 factors were found to be significant predictors for NAFLD using binary logistic regression modeling. Furthermore, the CPI and C-Peptide represent high diagnostic value for NAFLD in T2DM patients.

This study provides a more comprehensive risk factor analysis for NAFLD in T2DM patients. These data can be used to provide timely diagnosis and effective management of NAFLD.

## 1 Introduction

Non-alcoholic fatty liver disease (NAFLD) is defined by the presence of hepatic steatosis in the absence of secondary causes, and it is now recognized as the most common cause of chronic liver disease worldwide with a global prevalence of 25% among the general population.^[1]^ The prevalence of NAFLD in China has doubled in the past 20 years.^[2]^ However, it is usually overlooked in clinical practice.

Clinically, patients with NAFLD are usually asymptomatic, and with a history of smoking and unhealthy.^[3]^ Many recent studies have been performed to assess the association between NAFLD and metabolic syndromes (MetS), including diabetes with raised fast plasma glucose, abdominal obesity, hypertension, and hyperlipidemia, and they showed that MetS was significantly associated with NAFLD.^[4]^ Among these diseases, type 2 (T2DM) is believed to be the strongest predictor of NAFLD progression.^[2]^ As the amount of T2DM cases increases worldwide, the prevalence of NAFLD has increased proportionately. The presence of T2DM seems to accelerate the course of liver disease in NAFLD. Both the high prevalence of NAFLD in patients with T2DM and its serious clinical implications are cause for concern. Previous findings showed that the prevalence of NAFLD and nonalcoholic steatohepatitis (NASH) in patients with T2DM reached 60%,^[5]^ while the incidence rate of NAFLD among T2DM patients from 60 hospitals in southern China was 45.4%.^[6]^ In this study, the prevalence of NAFLD among patients with T2DM was found to be over 50%. According to previous reports, NAFLD is an independent risk factor for T2DM and cardiovascular disease, suggesting the association between NAFLD and T2DM may be bidirectional.^[7,8]^

With the steadily increasing prevalence of T2DM, the incidence of NAFLD in T2DM patients has become an important concern and imposes the need for early identification of patients who are at an increased risk for developing progressive liver disease. Recently, the routine methods for detecting NAFLD and its progress to fibrosis include liver biopsy, grayscale and Doppler sonography, fibroscan (FGFL) and computerized tomography (CT) scan, and magnetic resonance imaging (MRI). Doppler sonography examination has emerged as the noninvasive imaging method of choice for evaluation of liver blood supply and some of liver parenchyma's disorders.^[9]^ In addition, FGFL is another noninvasive test, performed by experienced medical doctors, to quantify liver fibrosis based on using ultrasound waves. However, abdominal US lacks the sensitivity required for mild steatosis analysis and requires significant knowledge and experience in analyzing the various ways NAFLD can present. As a result, the actual prevalence of NAFLD among T2DM patients is typically underestimated. Furthermore, computed tomography requires limited radiation exposure. Because of these limitations, liver biopsy remains the gold standard for the diagnosis and staging of NAFLD.^[10]^ However, an invasive liver biopsy is not an ideal diagnostic test for such a prevalent condition, as it is costly and carries the risk of iatrogenic complications, including pain, infection, bleeding, and even death.^[11]^ In addition, liver biopsy has some disadvantages such as significant sampling errors, interobserver variability, and the absence of diagnostic consensus. It is not a practice tool for regular screening of a patient with increased risk of NAFLD and advanced fibrosis.^[12]^ Therefore, routine screening for NAFLD remains a challenge, largely due to the lack of accurate non-invasive diagnostic techniques. The development of reliable noninvasive methods for the assessment of NAFLD is essential to guide the treatment of T2DM patients.

Recently, various serum biomarkers and laboratory tests have been proposed to screen for NASH in order to avoid redundant liver biopsies. These biomarkers include indicators of insulin resistance (IR), oxidative stress, inflammation, and apoptosis, as well as hormones and hepatic fibrosis markers.^[10]^ However, testing these markers can be costly, and thus their widespread use has been limited. Therefore, the development of reliable risk factors for NAFLD has become essential to guide therapy in patients with T2DM.

While many studies have identified T2DM as an independent risk factor for NAFLD, few studies have reported the risk factors of incident NAFLD in patients with T2DM. Therefore, this study sought to assess the prevalence and risk factors of NAFLD in patients with T2DM in China. Using the information obtained during routine pre-admission screening, including demographic, body measurement, renal chemistry, liver chemistry, serum lipid tests, and diabetes testing profile for T2DM patients, the most important predictors of NAFLD were investigated in T2DM patients. We expect that this study will provide important information for the early detection and proper control of NAFLD, and reduce the risk of developing NAFLD for T2DM patients.

## 2 Materials and methods

### 2.1 Study population

This is a retrospective, cross-sectional study among 2405 patients with T2DM treated and admitted for glucose control into the Endocrinology Department of the First Affiliated Hospital of Anhui University of Chinese Medicine (Anhui, China) from April 2017 to March 2019. The target patients were screened according to our exclusion criteria. The following diagnostic criteria based on the WHO diagnostic criteria of diabetes mellitus set in 1998 were used: fasting blood glucose ≥7.0 mmol/L, or typical symptoms of diabetes mellitus with random blood glucose ≥11.1 mmol/L, or 2 h blood glucose in glucose tolerance test ≥11.1 mmol/L.^[13]^ The exclusion criteria for this study were as follows:

1. patients who were more than 74 years old;
2. patients who excessively drank alcohol or had missing data in alcohol consumption habits;
3. patients with viral hepatitis, alcoholic hepatitis, drug-induced hepatitis, or autoimmune hepatitis;
4. patients who had taken hormones or lipid-regulating drugs in the last 3 months;
5. patients with cholecystitis or gallstones;
6. patients who recently had an infection, surgery, tumor, diabetic ketoacidosis, or blood disease;
7. patients who had hyperthyroidism or hypothyroidism;
8. patients with hyperuricemia, hypoproteinemia, gout, gestational diabetes, chronic kidney disease, or chronic gastritis;
9. patients with incomplete or missing data from their physical examination, laboratory tests, and liver US or CT (Fig. 2).

The study was approved by the Ethics Committee of the Affiliated Hospital of Anhui University of Chinese Medicine (Anhui, China).

### 2.2 Measurements

Three hundred eighty-four target patients were divided into two groups: NAFLD patients (study group) and non-NAFLD patients (control group). Totally 34 factors associated with NAFLD as independent variables were included in this study, and the demographic details, body measurements, and laboratory biochemical results were acquired from patient medical records. The demographic information included age, gender, duration of diabetes, drug history, medication history, smoking status, and alcohol consumption status. The smoking status of the patients was classified as non-smoker or current smoker. The physical examination data included weight, height, waist circumference, hip circumference, heart rate, and blood pressure. Routine laboratory tests, including renal chemistry, liver chemistry, blood lipids, and diabetes tests were collected from the admission and discharge records. More specifically, the serum biochemical factors mainly included fasting plasma glucose (FPG), hemoglobin a1c (HbA1c), fasting C-Peptide, fasting insulin (FINS), total cholesterol (TC), triglycerides (TG), high-density lipoprotein cholesterol (HDL-c), serum uric acid (SUA), aspartate aminotransferase (AST), alanine aminotransferase (ALT), as well as others. All the above information was obtained from the hospital database.

Blood samples were collected after overnight fasting for analysis. HbA1c was measured by ion exchange high performance liquid chromatography (Bio-Rad, VARIANTII TURBO, CA). The fasting samples for FINS and C-Peptide were collected and analyzed using a chemiluminescence kit (Autobio, Zhengzhou, China). FPG level was measured using the glucose oxidase method. Renal chemistry, liver chemistry, and blood lipids were determined using the liquid enzymatic method with an automatic biochemical analyzer (H7600; Hitachi Inc, Tokyo, Japan).

In addition, body mass index (BMI) was calculated as: BMI = body weight (kg)/height (m^2^); waist-to-hip ratio (WHR) was calculated as: WHR = Waist Circumference (m)/Hip Circumference (m); waist-to-height ratio ratio (WHtR) was calculated as: WHtR = Waist Circumference (m)/height (m); the homeostasis model assessment–IR (HOMA-IR) was calculated using the following formula: [FINS (μIU/mL) × FPG (mmol/L)]/22.5.

### 2.3 Definitions

In this study, the diagnostic criteria of NAFLD were based on the guidelines for the diagnosis and treatment of NAFLD set in 2010.^[14]^ The specific evaluation criteria of NAFLD were:

1. no history of drinking or alcohol consumption <140 g/week for men and 70 g/week for women, respectively;
2. transabdominal ultrasonography findings conformed to two of the following three manifestations:
  (a) the near-field echo in hepatic region (bright) was enhanced in a diffused mode compared with kidney;
  (b) intrahepatic duct had an unclear structure;
  (c) the far-field echo was attenuated;
3. presence of specific diseases that can cause fatty liver disease, including but not limited to viral hepatitis, medicated liver disease, total parenteral nutrition, hepatolenticular degeneration, and autoimmune liver disease.

### 2.4 Statistical analysis

Data were analyzed using the statistical software program, SPSS 22.0 (SPSS Inc, Chicago, IL). Continuous variables are expressed as mean ± SD, and were analyzed using an independent-sample *t* test. Univariate analysis was used to select the independent variables associated with the presence of NAFLD (*P* < .05). Subsequently, the binary logistic regression model was used to estimate the importance of examined variables associated with NAFLD. The odds ratios and 95% confidence intervals were calculated. *P* < .05 was considered statistically significant. Finally, all predictors were assessed using receiver operating characteristic (ROC) curves.

## 3 Results

### 3.1 Demographic and clinical features of participants

**Figure 1:**
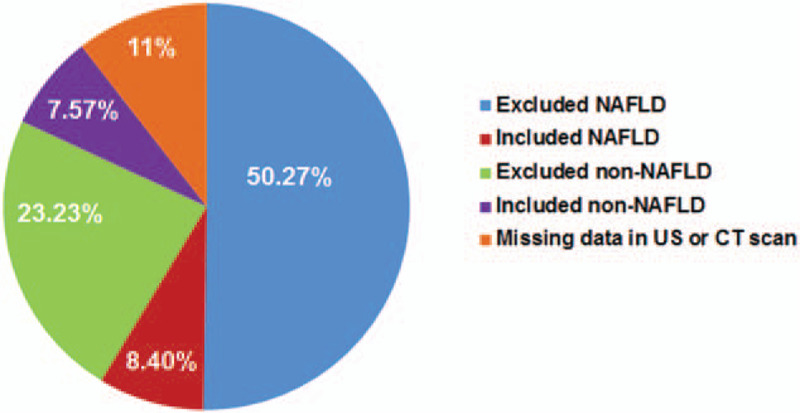
The prevalence of NAFLD among T2DM patients. Red represents included NAFLD patients; Blue represents excluded NAFLD patients; Green represents excluded non-NAFLD patients; Purple represents included non-NAFLD patients; Orange represents patients with missing data in their US or CT scan.

**Figure 2:**
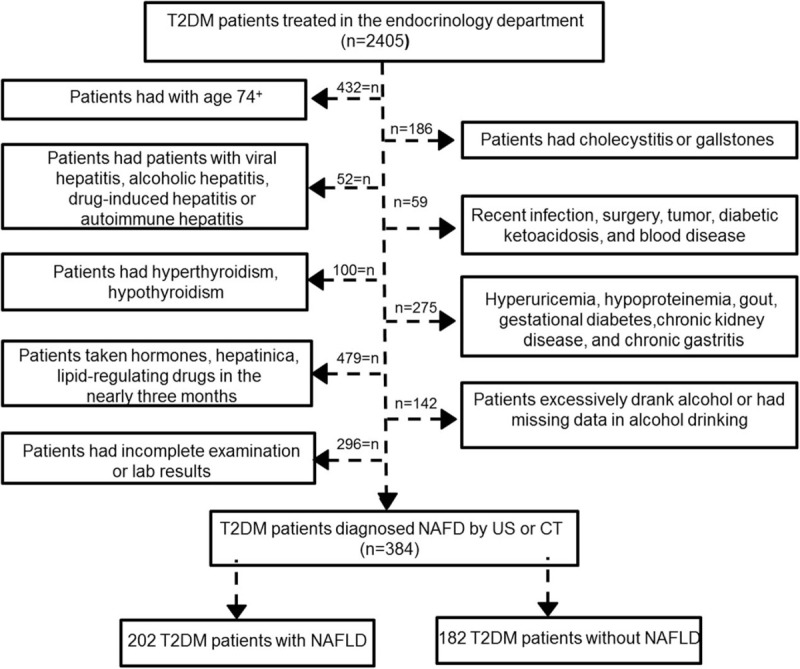
Study design and study population.

A total of 2405 patients with T2DM were involved in this study. Based on US imaging or CT scan, the prevalence of NAFLD was found to be over 58.67% in these patients (Fig. 1). As shown in the study flow diagram (Fig. 2), 146 patients were excluded for known liver disease, 279 for excessive alcohol intake and 289 due to the presence of hepatitis B surface antigen, and finally 384 patients were included in the analysis. These patients were divided into two groups: NAFLD patients (study group) and non-NAFLD patients (control group).

The demographic profile and clinical features of participants in the study and control groups are summarized in Table 1. For continuous variables, 7 factors including serum creatinine, TG, AST, ALP, GGT, fasting insulin, and HOMA-IR displayed a skewness >3. Subsequently, the log transformation was applied to these factors. Univariate analysis identified 17 parameters at enrollment that were significantly correlated with NAFLD (*P* < .05), and were entered into the binary logistic regression model. Our results showed that T2DM patients with NAFLD were younger than those without NAFLD (mean age 54.35 ± 10.77 years vs 57.15 ± 9.17 years, respectively). Interestingly, there was a significant association between age and having NAFLD was detected as78.37% of patients who were in <40 years age group had NAFLD (*P* < .001); younger individuals showed a stronger association between T2DM and NAFLD. Furthermore, T2DM patients with NAFLD had a higher BMI, WHR, WHtR, diastolic pressure, SUA, TG, ALT, AST, GGT, TP, albumin, fasting C-Peptide, and fasting insulin levels, but a lower low-density lipoprotein cholesterol (LDL-c) than those without NAFLD. Moreover, T2DM patients higher than 25 BMI had significantly more percentage of NAFLD (71.82%). In NAFLD group, 130 (64.36%) patients had BMI ≧ 25 and 72 (35.64%) patients had BMI < 25. In addition, HOMA-IR was higher in NAFLD patients than in non-NAFLD patients. However, mean duration of diabetes was shorter in patients with NAFLD than those without NAFLD (5.83 ± 5.58 years vs 7.92 ± 6.68 years).

**Table 1:** Demographic and clinical characteristics of T2DM patients with or without NAFLD.

Parameter	Overall (n = 384)	With NAFLD (n = 202)	Without NAFLD (n = 182)	*P*
Demographic				
Age (year)	55.64 (±10.12)	54.28 (±10.75)	57.15 (±9.17)	.005^∗∗^
<40	37 (100)	29 (78.37)	8 (21.62)	
41–50	73 (100)	37 (50.68)	36 (49.32)	
51–60	140 (100)	73 (52.14)	67 (47.86)	
>60	134 (100)	63 (47.01)	71 (52.99)	
Sex (male/female)	201/183	110/92	91/91	.384
Diabetic Duration (year)	6.74 (±6.10)	5.71 (±5.31)	7.92 (±6.68)	.001^∗∗^
Smoking status (yes/no)	54/330	31/171	23/159	.531
Body measurement				
Systolic Pressure (mm Hg)	131.08 (±17.00)	132.65 (±17.07)	129.35 (±16.79)	.057
Diastolic Pressure (mm Hg)	82.89 (±10.61)	84.57 (±10.45)	81.02 (±10.51)	.001^∗∗^
Heart rate (beats/min)	78.95 (±9.36)	79.60 (±9.34)	78.24 (±9.35)	.154
WHR	91.95 (±6.48)	93.54 (±63.03)	90.19 (±62.27)	<.001^∗∗∗^
WHtR	55.15 (±5.98)	56.85 (±55.19)	53.26 (±59.15)	<.001^∗∗∗^
BMI (kg/m^2^)	24.77 (±3.61)	26.07 (±3.61)	23.33 (±3.01)	<.001^∗∗∗^
<25	203 (100)	72 (34.47)	131 (64.53)	
≧25	181 (100)	130 (71.82)	51 (28.18)	
Renal chemistry				
Blood urea nitrogen	5.77 (±1.80)	5.73 (±1.59)	5.84 (±2.02)	.534
Serum creatinine^1^	58.35 (±26.22)	58.50 (±24.76)	58.12 (±27.82)	.909
Blood uric acid (μmol/L)	284.98 (±73.67)	302.39 (±67.64)	265.64 (±75.63)	<.001^∗∗∗^
Liver chemistry				
ALT (IU/L)	23.19 (±14.69)	27.76 (±17.15)	18.16 (±9.05)	<.001^∗∗∗^
AST (IU/L)^1^	18.73 (±8.69)	20.50 (±10.41)	16.77 (±5.66)	<.001^∗∗∗^
GGT (IU/L)^1^	33.85 (±38.96)	39.28 (±32.51)	27.84 (±44.38)	<.001^∗∗∗^
ALP (IU/L)^1^	98.22 (±40.21)	99.77 (±34.42)	96.40 (±45.79)	.164
Total bilirubin (μmol/L)	12.56 (±5.95)	12.74 (±6.56)	12.45 (±5.35)	.739
Direct bilirubin (μmol/L)	3.90 (±1.75)	3.88 (±1.81)	3.91 (±1.68)	.840
Indirect bilirubin (μmol/L)	8.65 (±4.78)	8.76 (±5.21)	8.52 (±4.26)	.620
Total protein (g/L)	68.52 (±6.18)	69.32 (±5.98)	67.64 (±6.27)	.008^∗∗^
Albumin (g/L)	42.13 (±4.12)	42.84 (±3.58)	41.36 (±4.52)	<.001^∗∗∗^
Globulin (g/L)	26.48 (±4.30)	26.62 (±4.08)	26.32 (±4.52)	.489
Albumin to globulin ratio	1.64 (±0.34)	1.65 (±0.36)	1.62 (±0.31)	.389
Blood lipids				
Triglyceride (mmol/L)^1^	2.14 (±2.50)	2.71 (±3.09)	1.51 (±1.37)	<.001^∗∗∗^
Cholesterol (mmol/L)	4.65 (±1.11)	4.75 (±1.18)	4.54 (±1.01)	.069
HDL-cholesterol (mmol/L)	1.11 (±0.34)	1.04 (±0.30)	1.20 (±0.36)	<.001^∗∗∗^
LDL-cholesterol (mmol/L)	2.82 (±0.88)	2.88 (±0.89)	2.76 (±0.87)	.171
Diabetes tests				
HbA1c (%)	8.50 (±2.21)	8.52 (±2.11)	8.47 (±2.31)	.817
FPG (mmol/L)	8.79 (±3.40)	9.07 (±3.32)	8.48 (±3.47)	.088
Fasting C-peptide (ng/mL)	2.26 (±1.12)	2.61 (±1.20)	1.87 (±0.88)	<.001^∗∗∗^
Fasting insulin (μIU/mL)^1^	9.52 (±11.11)	12.46 (±13.67)	6.27 (±5.79)	<.001^∗∗∗^
HOMA-IR^1^	3.89 (±7.00)	5.24 (±9.02)	2.40 (±2.95)	<.001^∗∗∗^

Continuous variables with normal distribution (mean ± sd) were analyzed using independent-sample *t* tests. ALP = Alkaline phosphatase, ALT = alanine aminotransferase, AST = aspartate aminotransferase, GGT = Gamma glutamyl transferase, HbA1c = Glycated hemoglobin, HbA1c = Glycated hemoglobin. ^1^Log transformation was applied to this factor before including in *t* tests.

∗∗ *P* < .01.

∗∗∗ *P* < .001.

### 3.2 Personal and clinical factors associated with NAFLD in 2TDM patients

**Figure 3:**
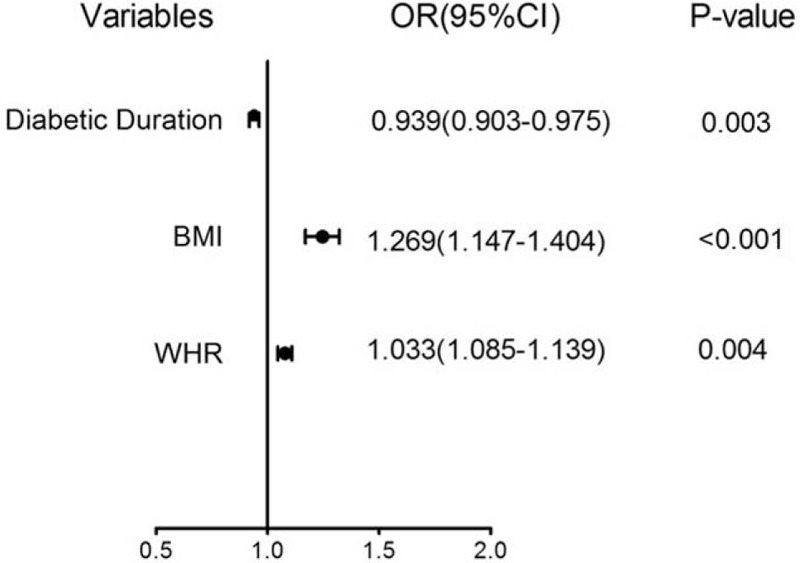
Forest plot of each predictor. The left column lists the predictors. The odds ratio for each of these factors is represented by a *circle*, and confidence intervals are represented by *horizontal lines*. BMI = body mass index, CI = confidence interval, OR = odds ratio, WHR = waist hip rate.

We performed binary logistic regression analysis to determine the personal and clinical factors associated with NAFLD. As shown in Table 2, the risk factors for NAFLD were BMI and WHR, while diabetic duration was a protective factor (odds ratio 0.939, 95% CI 0.902–0.979; *P* < .01). Compared with WHR, increased BMI was associated with higher risk of NAFLD among T2DM patients (odds ratio 1.241, 95% CI 1.118–1.378; *P* < .001). However, diastolic pressure, age, and WHtR did not have a significant influence. The forest plot of each predictor is shown in Figure 3.

**Table 2:** Personal and clinical factors associated with NAFLD among T2DM patients.

Parameter	B coefficient	Odds ratio	95% CI	*P*
Age (year)	−0.016	0.985	0.960–1.010	.230
Diabetic duration (year)	−0.062	0.939	0.902–0.979	.003^∗∗^
Diastolic (mm Hg)	0.020	1.020	1.020–0.998	.074
WHR (%)	0.073	1.076	1.024–1.130	.004^∗∗^
WHtR (%)	−0.001	0.999	0.934–1.068	.982
BMI (kg/m^2^)	0.216	1.241	1.118–1.378	<.001^∗∗∗^

95% CI = 95% confidence interval, BMI = body mass index, NAFLD = non-alcoholic fatty liver disease, T2DM = type-2 diabetes mellitus, WHR = waist hip rate, WHtR = waist-to-height ratio.

∗∗ *P* < .01.

∗∗∗ *P* < .001.

### 3.3 Biochemical factors associated with NAFLD in 2TDM patients

**Figure 4:**
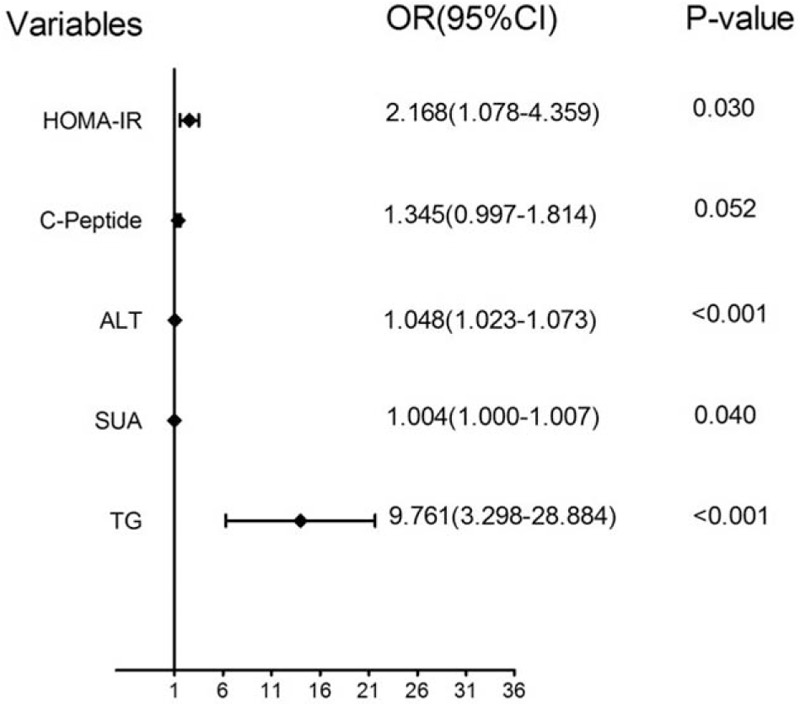
Forest plot of each biochemical predictor. The left column lists the predictors. The odds ratio for each of these studies is represented by a *diamond*, and confidence intervals are represented by *horizontal lines*. ALT = alanine aminotransferase, CI = confidence interval, HOMA-IR = homeostasis model assessment of insulin resistance, OR = Odds Ratio, SUA = serum uric acid, TG = triglyceride.

Binary logistic regression (Backward Elimination [Wald] method) was performed to estimate the independent biochemical risk factors for NAFLD after adjustments. The combined predictive indicator (CPI) was also obtained. Logistic regression analysis revealed that NAFLD was positively associated with TG, ALT, and SUA levels. The forest plot of each predictor is shown in Figure 4.

**Figure 5:**
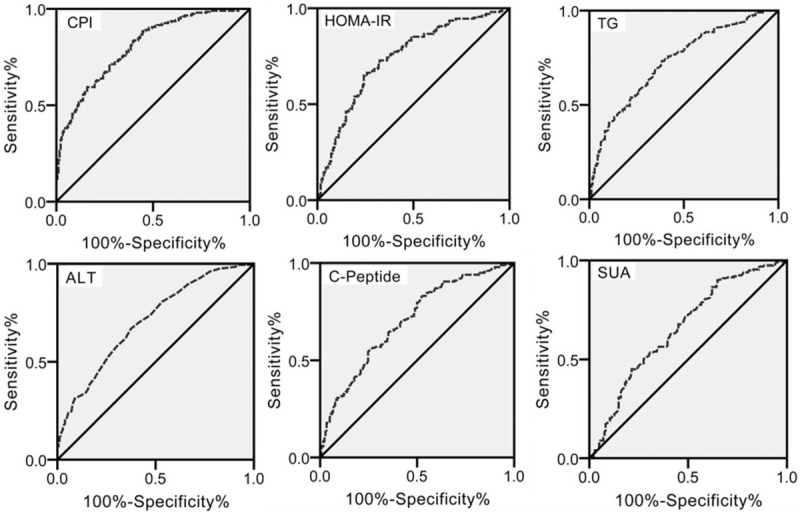
The ROC curve of biochemical predictor and CPI. Sensitivity measurements are on the *y*-axis, and 1-specificity is on the *x*-axis. The area under the curve represents the prediction accuracy. The optimal cutoff for each predictor was defined using the SPSS software. ALT = alanine aminotransferase, CPI = combined predictive indicator, HOMA-IR = homeostasis model assessment of insulin resistance, SUA = serum uric acid, TG = triglyceride.

Finally, the ROC curve was used to evaluate each biochemical predictor and the CPI. The ROC curves (Fig. 5) for SUA, ALT, TG, C-Peptide, HOMA-IR, and CPI displayed area under the curve (AUC) values of 0.657, 0.705, 0.729, 0.702, 0.740, and 0.805, respectively. An AUC value between 0.7 and 0.8 is acceptable and a value >0.8 represents high diagnostic value for NAFLD. Consequently, CPI is more helpful for the diagnosis of NAFLD than any of the other biochemical predictors. As shown in Table 4, the sensitivity and specificity of CPI were 71.3% and 72.5%, respectively.

## 4 Discussion

NAFLD, as detected by US or CT, is common in patients with type-2 diabetes. This study showed that NAFLD was found among 2045 T2DM patients at a prevalence of 58.67%. Furthermore, we collected the clinical and biochemical information of patients with type 2 diabetes, and investigated significant NAFLD risk factors. We found that the most significant risk factors for NAFLD in both univariate and multivariate level analyses were BMI, WHR, TG, UA, ALT, and IR. In addition, the duration of diabetes in T2DM patients with NAFLD was shorter than that among people without NAFLD (Table 1). The logistic regression analysis revealed that the duration of diabetes was a protective factor for NAFLD (Table 2 and Fig. 3). These results are similar to those found by Takeuchi Y,^[15]^ indicating that the presence of NAFLD is enhanced by the development of T2DM.

A strong association between obesity and NAFLD has been reported,^[4]^ which is particularly concerning given the raising rates of obesity. Our results are largely consistent with those reported previously which showed that BMI, WHR, and WHtR are anthropometric measurements widely used to evaluate the effects of obesity on NAFLD.^[16–18]^ Compared with the control group, BMI, WHR, and WHtR were significantly higher in T2DM patients with NAFLD. In addition, logistic and ROC curve analyses revealed that BMI and WHR were effective prognostic indicators of NAFLD. Our results did not include WHtR as a significant prognostic factor, however Lin et al^[16]^ observed a strong correlation between WHtR and the severity of pediatric NAFLD. In addition, increased WHtR was reported to significantly correlate with increased cardiometabolic risk factors,^[19]^ hypertension,^[20]^ and mortality.^[21]^ One recent study found that individuals with NAFLD showed greater improvements in liver function and insulin sensitivity after moderate, diet-induced weight loss than individuals without NAFLD.^[22]^ Above all, this study highlights the importance of including weight management in treatment plans for T2DM patients with NAFLD.

Dyslipidemia is a major risk factor for NAFLD, and high serum triglyceride levels may be a clue to the presence of NASH.^[23,24]^ In this study, TG and HDL-cholesterol levels were significantly different between T2DM patients with and without NAFLD (Table 1). However, only a high TG level remained a highly significant predictor of NAFLD after binary logistic regression analysis (Table 3, Fig. 4). Our results suggest that serum triglyceride level is more strongly associated with NAFLD than HDL-cholesterol, however, another study in T2DM patients in Abha City found that NAFLD had a stronger association with HDL-cholesterol than TG levels.^[25]^ The same results were found in studies conducted by researchers in Poland.^[26]^ This discrepancy may be explained by differences in race, and fluctuations in the level of HDL-cholesterol.

**Table 3:** Routine blood biochemical factors associated with NAFLD among T2DM patients.

Parameter	B coefficient	S.E.	Wald	OR	95% CI	*P*
TG (mmol/L)^1^	2.278	0.554	16.942	9.761	3.298–28.884	<.001^∗∗∗^
SUA (μmol/L)	0.004	0.002	4.202	1.004	1.000–1.007	.040^∗^
ALT (IU/L)	0.047	0.012	14.941	1.048	1.023–1.073	<.001^∗∗∗^
C-peptide (ng/mL)	0.296	0.153	3.765	1.345	0.997–1.814	.052
HOMA-IR^1^	0.774	0.356	4.709	2.168	1.078–4.359	.030^∗^

95% CI = 95% confidence interval, ALT = alanine aminotransferase, HR = waist hip rate, WHtR = waist-to-height ratio. ^1^ Log transformation was applied to this factor before including in t tests.

∗ *P* < .05.

∗∗∗ *P* < .001.

Liver chemistry tests are the most widely used indicators of liver inflammation or liver cell damage. According to previous studies, increased AST and ALT levels indicate hepatocellular injury, and increased ALP indicates cholestatic injury.^[27,28]^ Additionally, serum gamma glutamyl transferase (GGT) has been considered a surrogate marker of NAFLD-induced oxidative stress and hepatocellular damage.^[29]^ Our univariate analysis revealed that T2DM patients with NAFLD displayed significantly higher levels of total protein, albumin, ALT, as well as natural log of AST, ALP, and GGT than T2DM patients without NAFLD (Table 1). However, only ALT values were positively correlated with NAFLD in multivariate level (odds ratio 1.048; 95% CI 1.023–1.073; *P* < .001). Our results are largely consistent with previous studies that reported ALT as a significant independent factor associated with a higher risk of developing NAFLD.^[30]^ Interestingly, our logistic regression analysis failed to find a significant association between NAFLD and high levels of AST. A similar association has been reported in previous studies.^[25]^ This difference may be due to the fact that AST is generally lower than the ALT level in NAFLD patients.^[28]^

It is well known that T2DM and NAFLD share a common pathogenic mechanism of IR.^[31,32]^ NAFLD can induce or aggravate IR in patients. Here, we calculated the HOMA-IR index, which is a marker of IR. HOMA-IR and HOMA-B indices are useful for predicting incident NAFLD. Several prospective studies have shown that HOMA-IR levels in T2DM patients with NAFLD are significantly higher than those in T2DM patients without NAFLD.^[33,34]^ These results support our finding that HOMA-IR is an independent risk factor for NAFLD in T2DM patients. Our analysis revealed that the cut-off point of HOMA-IR to assess the risk of incident NAFLD in T2DM patients was 2.67, and ROC curve analysis was performed to determine this value. The AUC (95% CI) for HOMA-IR was 0.740, indicating that it may serve as a powerful predictor for NAFLD in T2DM patients.

In addition to IR, C-Peptide has been associated with many risk factors for NAFLD including cardiovascular diseases and metabolic syndrome. According to previous studies, C-Peptide levels are increased in patients with NASH.^[35,36]^ However, there is limited evidence connecting NAFLD and C-Peptide levels at multivariate level in T2DM patients. In this study, for every unit increase in C-Peptide, the risk of NAFLD in T2DM patients increased by 1.345-fold, and the AUC (95% CI) of C-Peptide was 0.702, suggesting that fasting C-Peptide level may be a powerful predictor for NAFLD in T2DM patients. In addition, logistic regression analysis revealed that C-Peptide, not insulin, is an independent risk factor of NAFLD, which may indicate that NAFLD has a stronger association with C-Peptide than insulin. However, the application of C-Peptide as a biomarker for therapies designed to improve insulin sensitivity remains to be determined.

T2DM can be diagnosed directly from a patient's HbA1c level (≧6.5%); however, we did not observe any association between the presence of NAFLD and HbA1c, suggesting that HbA1c levels are not a suitable predictor of NAFLD in T2DM patients. This is consistent with results from previous studies.^[25,26]^ Another study did note that a trend of increased HbA1c level was observed among diabetic patients with NAFLD.^[37]^

As previously reported,^[38]^ SUA level in T2DM patients with NAFLD was positively correlated with central obesity, abnormal liver enzymes, abnormal lipid metabolism, and glucose metabolism, which suggests the clinical importance of SUA in T2DM patients with NAFLD. In agreement with the study described above, the results of our study revealed that SUA was significantly higher in T2DM patients with NAFLD compared to those without NAFLD, suggesting SUA is an independent risk factor for the development of NAFLD. According to previous reports, elevated SUA is closely related to IR.^[39]^ In addition, increased IR led to increased levels of glucose metabolites, such as ribose-5-phosphate and pyrophosphate, and eventually increased SUA levels.^[40]^ These results supported our findings that SUA level and IR play central roles in the development of NAFLD in T2DM patients.

Our results showed that NAFLD is associated with many biochemical factors, including renal chemistry (SUA), liver chemistry (ALT), serum lipid (TG), and diabetic effects (C-Peptide and HOMA-IR), suggesting that a combination of these factors is more significant in predicting NAFLD among T2DM patients. Therefore, the CPI was determined in this study. Results showed that the AUC value of the CPI was higher than any of the other individual biochemical predictors (Table 4 and Fig. 5). Our analysis indicated that the pathogenesis of NAFLD is the result of multiple factors, making the CPI value of great clinical significance for the diagnosis and treatment of NAFLD in T2DM patients.

**Table 4:** Operating characteristic curves of each continuous variable.

Parameter	AUC (95%CI)	S.E.	Sensitivity (%)	Specificity (%)	Cut off	*P*
TG (mmol/L)	0.729 (0.679–0.779)	0.025	61.5	73.3	1.41	<.001^∗∗∗^
SUA (μmol/L)	0.657 (0.602–0.712)	0.028	90.1	35.2	224.5	<.001^∗∗∗^
ALT (IU/L)	0.705 (0.654–0.756)	0.026	66.8	62.6	18.5	<.001^∗∗∗^
C-peptide (ng/mL)	0.702 (0.650–0.754)	0.026	82.7	47.8	1.72	<.001^∗∗∗^
HOMA-IR	0.740 (0.691–0.790)	0.025	65.3	75.3	2.67	<.001^∗∗∗^
CPI	0.805 (0.762–0.847)	0.022	71.3	72.5	0.528	<.001^∗∗∗^

ALT = alanine aminotransferase, CPI = combined predictive indicator, HOMA-IR = homeostasis model assessment of insulin resistance, SUA = serum uric acid, TG = triglyceride.

∗∗∗ *P *< .001.

This systematic study sought to report the prevalence and factors associated with NAFLD among T2DM patients in Anhui Provincial, China. However, this study has some limitations. The diagnosis of NAFLD was based on ultrasonographic imaging or CT scan examination, but was not confirmed by invasive biopsy diagnosis. Additionally, the differences in body fat distribution or fat metabolism between races and ethnicities may also affect the prevalence of NAFLD. We should note that the patients enrolled in this study belong to the Han Chinese ethnic group. Finally, this study lacked medication information for the patients with diabetes and some antidiabetic drugs may have an effect on NAFLD.

## 5 Conclusions

NAFLD is highly prevalent in T2DM patients. This study provides the most important associated factors (triglyceride, homeostasis model assessment of IR, body mass, waist hip rate, diabetic duration, C-Peptide, alanine aminotransferase, serum uric acid, and CPI) for NAFLD in T2DM patients. These data can be used for timely diagnosis and effective management of NAFLD, and can help minimize liver-related morbidity and mortality in the diabetic population.

## Acknowledgments

This study was supported by Nature Science Found of Anhui Province (No.1808085QH250), University Natural Science Foundation of Anhui (KJ2018A0290), National Natural Science Foundation of China (No. 82004139, 82004139, 81873139 and 81803938), Scientific and technological projects of Henan Province (202102310068), and Nature Science Found of Anhui University of Chinese Medicine (No.2018zryb48).

## Author contributions

**Conceptualization:** Qiumei Zhou, Kaiquan Huang, Xingxing Huo.

**Data curation:** Jiajia Wang, Wancun Zhang.

**Formal analysis:** Yulong Wang.

**Investigation:** Dehui Qi.

**Methodology:** Yating Liu, Tingting Li, Wei Yao.

**Software:** Hui Jiang.

**Writing – original draft:** Qiumei Zhou.

**Writing – review & editing:** Xingxing Huo.
